# Guided Growth of the Proximal Femur for the Management of the ‘Hip at Risk’ in Children with Cerebral Palsy—A Systematic Review

**DOI:** 10.3390/children9050609

**Published:** 2022-04-25

**Authors:** Moritz Lebe, Renée Anne van Stralen, Pranai Buddhdev

**Affiliations:** 1 Broomfield & Addenbrookes Hospitals, Chelmsford CM1 7ET, UK; moritz.lebe@nhs.net (M.L.); pranai.buddhdev@nhs.net (P.B.); 2 Erasmus MC Sophia Children’s Hospital, 3015 CN Rotterdam, The Netherlands

**Keywords:** guided growth, DDH, cerebral palsy, temporary medial hemiepiphysiodesis for the proximal femur (TMH-PF)

## Abstract

Background: Guided growth is frequently used to modify lower-limb alignment in children, and recently temporary medial hemiepiphysiodesis of the proximal femur (TMH-PF) has been used for the management of hips at risk of subluxation in cerebral palsy (CP) patients. The aim of our study was to evaluate the efficacy of TMH-PF in the management of neuromuscular hip dysplasia in children with cerebral palsy. Methods: A systematic search of the literature was performed by using PubMed, EMBASE, CINAHL, MEDLINE, Scopus and Cochrane databases. Pre- and postoperative radiographic changes of the migration percentage (MP), head-shaft angle (HSA) and acetabular index (AI) were included in a meta-analysis. Secondary outcomes were treatment complication rates, technical considerations and the limitations of this novel technique. Results: Four studies (93 patients; 178 hips) met the eligibility criteria for inclusion in the meta-analysis. All three radiographic measurements showed significant changes at a minimum of 2 years of follow-up. Mean changes for MP were 8.48% (95% CI 3.81–13.14), HSA 12.28° (95% CI 11.17–13.39) and AI 3.41° (95% CI 0.72–6.10), with I^2^ of 75.74%, 0% and 87.68%, respectively. The serious complication rate was overall low; however, physeal ‘growing off’ of the screw was reported in up to 43% of hips treated. Conclusion: TMH-PF is an effective and predictable method to treat CP patients with ‘hips at risk’, and the overall complication rate is low; however, further work is required to identify the best candidates and surgical timing, as well as choice of technique and implant.

## 1. Introduction

Coxa valga is a complex three-dimensional deformity of the proximal femur, caused by altered growth of the proximal femoral physis [1,2,3]. Its cause can be idiopathic; projectional on plain radiographs, due to femoral anteversion; or in association with a variety of conditions, including developmental dysplasia of the hip (DDH), Charcot–Marie–Tooth (CMT) disease or cerebral palsy (CP) [4,5]. In CP patients, coxa valga, which is related to excessive anteversion, is commonly related to the functional status of the patient, in combination with muscle spasticity and weakness, and subsequent contractures can lead to symptomatic, progressive hip joint subluxation and dislocation, causing disturbed seated balance or standing abilities, difficulty with perineal care, the development of decubitus ulcers and poor quality of life [6,7,8,9,10,11]. Up to one-third of children with CP have hip instability, with an increasing incidence associated with GMFCS level—>60% of GMFCS IV/V [12,13,14,15], as measured using the Reimers migration percentage [16].

Traditional surgical management, typically reserved for hips with a migration percentage of 40% or more, includes hip reconstruction involving soft tissue releases, femoral and pelvic osteotomies [17]. These procedures are associated with significant perioperative morbidity, including pain; increased blood loss; and lengthy anesthetic and inpatient recovery times, often complicated with peri-operative infections [18,19]. With improved surgical techniques, orthopedic implants and enhanced postoperative pathways, weightbearing can be resumed shortly after surgery; however, traditional treatment commonly included a period of non-weight-bearing, with some surgeons preferring to augment their reconstruction with a hip spica or abduction brace [20,21].

**Figure 1:**
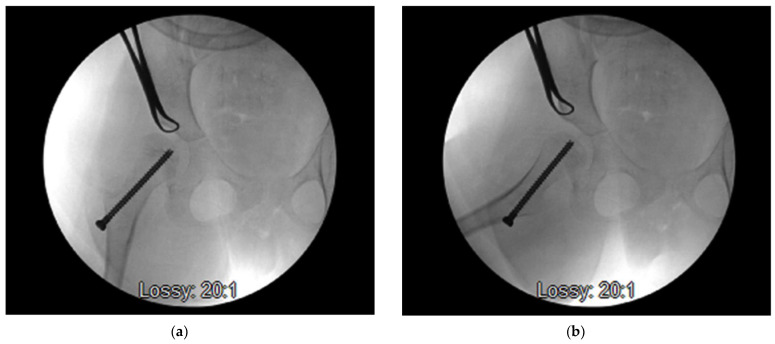
Final AP (**a**) and lateral (**b**) fluoroscopy image showing the desired screw placement across the proximal femoral physis (with copyright permission from Jon Davids [35]).

Guided growth procedures are well established in the treatment for the gradual correction of angular and rotational limb deformities in children [22,23,24,25]. Anterior hemiepiphysiodesis of the distal femur has been shown to be effective in the treatment of fixed flexion deformity of the knee when compared to traditional osteotomies [26,27,28,29,30]. Figure 1 shows intra-operative radiographs of this minimally invasive technique, which has been recently applied to the proximal femoral physis for various conditions [23,31,32,33,34,35]. By placing a screw over the physis on the medial side, the tethering that occurs on the medial side will result in progressive varus of the proximal femur. It is understood that this manipulation of the proximal femoral anatomy can alter the course of secondary acetabular dysplasia [36,37]. Furthermore, it is recognized that guided growth procedures of the proximal femoral physis can be carried out as day case procedures, require a shorter operating time and allow for immediate weight bearing/standing when performed in non-ambulatory patients.

This systematic review reports a quantitative summary of postoperative radiological outcome measures of temporary medial hemiepiphysiodesis for the proximal femur (TMH-PF) in children with CP. We also summarize the technical considerations, reported treatment complications and limitations of this novel intervention.

## 2. Materials and Methods

This systematic review was performed in accordance with the guidelines of the Cochrane Handbook for Systematic Reviews and the PRISMA-P statements [38]. The protocol followed was registered with and accepted by the International Prospective Register of Systematic Reviews (PROSPERO) on 15.01.2021 (CRD42021226864).

### 2.1. Information Sources and Search Terms

A comprehensive search of the literature was performed by using PubMed, MEDLINE, Cochrane, Embase and Scopus databases, and Level IV or higher original articles were selected for this review. Search terms, including Boolean operators suitable for each database, were (“guided growth” OR “hemiepiphysiodesis” OR “TMH”) AND (“coxa valga” OR “hip” OR “DDH” OR “pelvis” OR “prox* femur”). Cross-reference search results of the included studies and gray literature were included when available. The literature search was performed in January 2021. 

Our inclusion criteria were pediatric, skeletally immature patients with cerebral palsy, as described and updated by Bax et al. [39,40]; and a “hip at risk” of progressive subluxation, as described by Davids et al. and others [12,35]. 

Exclusion criteria were previous proximal femur or pelvis operations, case reports, technical notes, and published abstracts.

### 2.2. Selection Process

**Figure 2:**
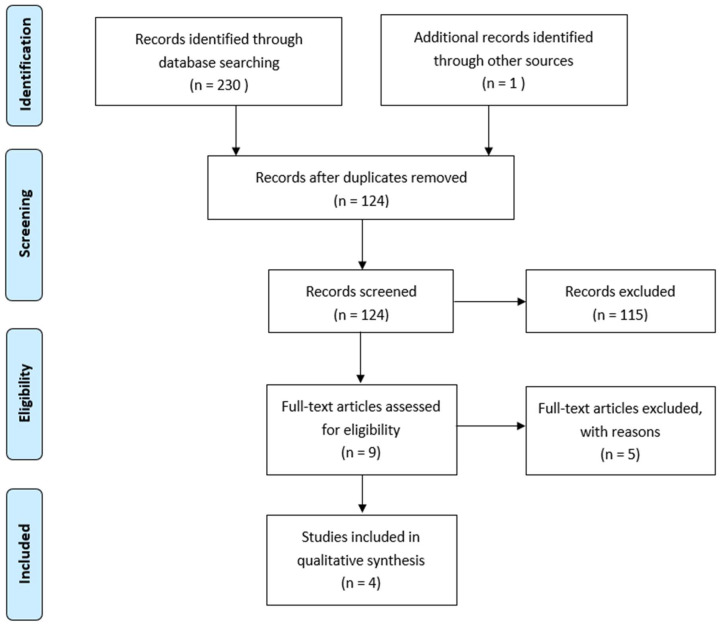
PRISMA flowchart demonstrating the results from the literature search and exclusions of papers.

The PRISMA flow chart is illustrated in Figure 2. Two independent reviewers (ML and PB) separately, and blinded to each other, conducted the screening of search results against the in/exclusion criteria based on title, abstract and keywords. Disagreements were resolved by an independent third author (RvS). After the removal of duplicates, 9 titles were selected for full-text review, of which 5 were excluded with reasons. Four articles were included for quantitative analysis.

### 2.3. Assessment of Quality and Bias

Risk of bias was assessed for all studies, using the ROBIN-I checklist [41], as recommended in the Cochrane Handbook for Systematic Reviews [42]. Findings are summarized in Table 1 and show the overall risk for bias as critical. All four studies had a small sample and were retrospective, with level IV case series and risk of bias due to confounding and selection of patients; moreover, the measurement of outcomes data remains a concern.

### 2.4. Outcome Measures and Statistics 

The primary outcome was a change of radiographic angles after at least 2 years of follow-up. Secondary outcomes were complication rates, as graded by the Clavien–Dindo System, as well as a qualitative analysis of technical considerations based on the included papers [43,44,45]. Meta-analysis was performed by using Stata (StataCorp. 2019. Stata Statistical Software: Release 16. StataCorp LLC, College Station, TX, USA). Changes in pre- and postoperative radiographic angles were evaluated by means and standard deviations (SDs), and heterogeneity tests were performed; the random-effect model was applied if heterogeneity existed.

**Table 1:** ROBINS-I: Risk of bias assessment of non-randomized trials.

Reference	Bias Due to Confounding	Bias in Selection of Participants	Bias in Classification of Intervention	Bias due to Deviations from Intended Intervention	Bias Due to Missing Data	Bias in Measurement of Outcomes	Bias in Selection of the Reported Results	Overall Risk of Bias
[32]	Critical	Critical	Low	Low	No information	Serious	Moderate	Critical
[34]	Critical	Critical	Low	Low	No information	Serious	Moderate	Critical
[46]	Critical	Critical	Low	Low	Low	Critical	Moderate	Critical
[47]	Critical	Critical	Moderate	Low	Low	Critical	Moderate	Critical

## 3. Results

Our literature search has identified *n* = 231 titles. After the removal of duplicates, *n* = 124 titles, abstracts and keywords were screened for inclusion. Nine articles underwent full-text review, of which *n* = 4 met our in-/exclusion criteria and were subsequently selected for quantitative analysis. All studies included were level IV retrospective case series; the study characteristics and outcomes are summarized in Table 2.

### 3.1. Primary Outcomes

Postoperative changes of radiographic measures after ≥2 years of follow-up are presented in Table 2, which describes patient characteristics, methods and outcome measures used by all studies [32,34,46,47] included in our analysis. Most commonly, changes in the migration percentage (MP), head/neck-shaft angle (HSA/NSA) and acetabular index (AI) were reported at one year, as well as at two years or last follow-up review and compared with preoperative measurements. Some authors have performed additional soft tissue releases, and in >95% of patients included (178 hips in 93 patients), guided growth procedures were performed bilaterally during one theater attendance. One author [47] has, in addition, performed a subgroup analysis to assess the influence of transphyseal screw position on femoral remodeling and physis growing off the screw, as well as relevant predictive factors for a postoperative decrease in HSA.

### 3.2. Quantitative Analysis

We have performed three random-effect meta-analyses to reflect the most commonly reported changes of mean radiographic angles after TMH-PF surgery (MP, HSA and AI). Hsu et al. [48] and Lee et al. [32] published 2 years of post-operative data, Portinaro et al. [34] reported 5 years of post-operative data, and Hsieh et al. [46] reported data with a mean follow-up of 50 months and a minimum of 2 years. To allow statistical analysis, we combined 2 years or more of published follow-up data in our analysis. 

#### 3.2.1. Migration Percentage

**Figure 3:**
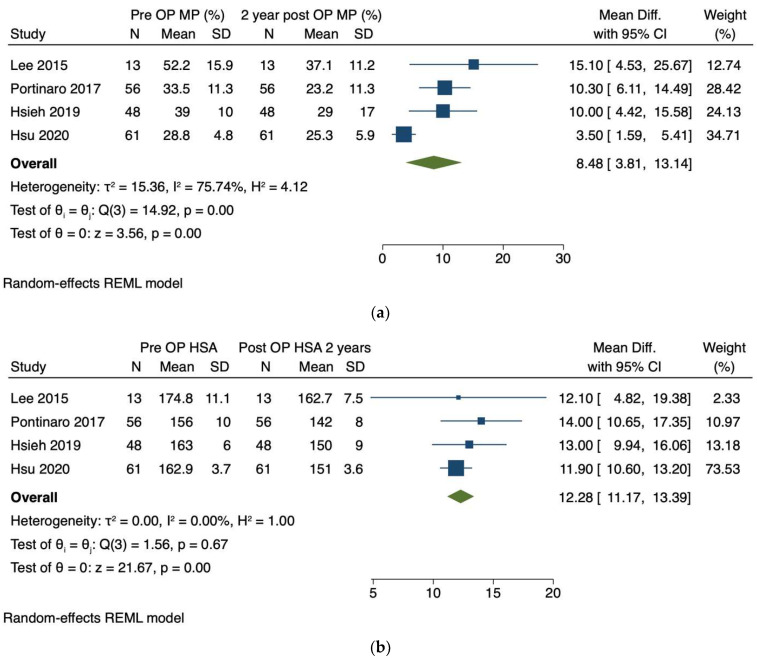

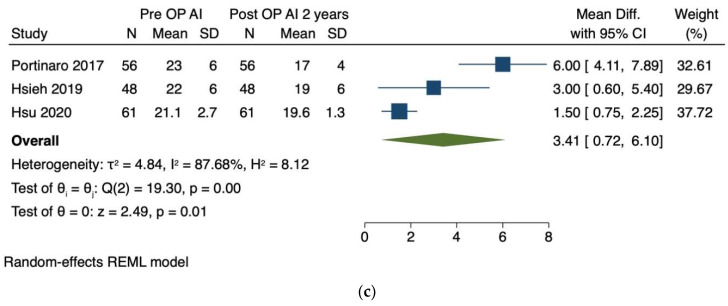
Forest plot demonstrating changes of primary outcomes measures after 2 and more years of follow-up. (**a**) Changes of migration percentage (MP). (**b**) Changes of head-shaft angle (HSA). (**c**) Change of acetabular index (AI).

The mean migration percentage was reported in *n* = 178 hips by four authors [32,34,46,47] preoperatively and at least 2 years post-operatively as 34.74% (SD 11.45) and 26.50% (SD 12.27), respectively. We identified a significant (*p* < 0.01) weighted mean difference of 8.49% (95% CI 3.81–13.14, Figure 3a), with an I^2^ of 75.7% and an average Hedges’s g effect size of 0.77 (95% CI 0.66–0.99). 

#### 3.2.2. Head-Shaft Angle

The mean head-shaft angle was also reported in *n* = 178 hips by four authors [32,34,46,47], with a mean preoperative HSA of 161.63° (SD 8.80) and 148.75° (SD 8.97) after at least 2 years of post-operative follow-up. We identified a significant (*p* < 0.01) weighted mean difference of 12.28° (95% CI 11.17–13.39, Figure 3b), an I^2^ heterogeneity of 0% and an average Hedges’s g effect size of 1.94 (95% CI 1.07–2.81). 

#### 3.2.3. Acetabular Index

The mean acetabular indices were also reported in *n* = 165 hips by three authors [34,46,47], with a mean preoperative AI of 22.0° (SD 5.07) and 18.54° (SD 4.19) after at least 2 years of postoperative follow-up. We identified a significant (*p* < 0.01) weighted mean difference of 3.41° (95% CI 0.72–6.10, Figure 3c), I^2^ heterogeneity was 87.68% and the average Hedges’s g effect size was 0.79 (95% CI 0.41–1.17).

### 3.3. Secondary Outcome-Reported Technical Considerations, Complications and Limitations of TMH-PF

Lee et al. [32] described the surgical technique used for TMH-PF. They placed the screw two or three threads across the physis and assessed for protrusion of the screw into the joint. In their cohort, there were no complications, including infection, femoral neck fractures, implant failure, chondrolysis or osteonecrosis. All screws, however, backed out of the femoral epiphysis between 1 and 2 years postoperatively as the child grew. They reported no significant changes of the HSA between 1 and 2 years, and in some cases, continuing varus deformation after backing out of the screw, indicating a potential premature partial physeal closure.

While describing their surgical technique, Portinaro et al. [34] emphasized placing the cannulated screw guidewire in the inferomedial quadrant of the proximal femoral growth plate in order for the tip of the screw to reach 2 to 3 mm under the bony contour of the femoral head. They highlighted the importance of preventing the protrusion of the k-wire and screw into the joint and utilized a 4.5 mm cannulated screw instead of a 6.5 or 7.0 mm cannulated screw. In 9/56 hips, the physis grew off the screw, of which two hips underwent screw replacement surgery; however, in one of these cases, the screw head of the initial screw broke. The subsequent screw revisions (*n* = 4) were performed by adding a second screw, rather than exchanging the initial screw. The remaining three cases required VDROs, and there were no other complications, such as AVN, chondrolysis, fractures or wound infections.

Hsieh et al. [46] described the ideal position of the screw, as aimed at the medial one-third of the capital epiphysis on the coronal plane and centered along the axis of the femoral neck on the lateral plane. Depending on the femoral size, 6.0 mm fully threaded or 7.0 mm partially threaded screws were used, aiming to pass at least three threads across the physis. They found that the physis grew off the screw in 21 of 48 hips (43%), 15 of 48 hips underwent a replacement with a longer screw, and 8 hips in 5 patients underwent subsequent reconstructive surgery, such as VDROs. They concluded that this technique offers predictable results if the migration percentage is under 50% and there is enough growth remaining. They recommended restricting its use in patients with a migration percentage over 50%.

With respect to technical considerations, Hsu et al. [47] mention that the optimal position of the screw remains unclear. In order to prevent iatrogenic injury to the growth plate, repositioning was not attempted once a transphyseal position was achieved through the medial physis. They described 16 cases of the physis growing off the screw, and younger age at the time of surgery was identified as a significant risk factor (mean age of 7 years comparted to 9 years). Furthermore, it was suggested that medial positioning of the screw increases the risk of physeal growing off; this might only be appropriate for older children with less remaining growths. Describing only a limited improvement in the acetabular index at the 2-year follow-up, they concluded that the effect of guided growth on the acetabular development might be limited.

## 4. Discussion

Temporary medial hemiepiphysiodesis of the proximal femoral physis (TMH-PF) is a relatively novel surgical technique that was first reported in an animal model [36,37]. It has since been successfully performed in pediatric patients with coxa valga, due to type II AVN in DDH [49], and in patients with cerebral palsy with ‘hips at risk’ [47]. This study is the first systematic review and meta-analysis to summarize postoperative radiographic changes, complications and revision rates. We found significant changes in the migration percentage, head-shaft angle and acetabular index after at least 2 years of follow-up, with a mean difference of 8.48%, 12.28 degrees and 3.41 degrees, respectively. Growing off of the screw can be classified as a grade IIIb complication according to the modified Clavien–Dindo System and occurs in 15–50% of cases, whereas progressive hip subluxation (failure of treatment) needing invasive osteotomies, was reported in 5 to 21% of cases [34,45]. Several factors, including age, screw position, growth potential of the capital physis and level of gross motor function, are understood to influence the individual amount and velocity of anatomic changes of the proximal femur, and it remains unclear to what extent guided growth moderates those changes [50,51]. The possibility of coxa vara overcorrection due to physeal injury also requires further investigation [37].

Davids [35] has published a detailed technical summary of TMH-PF and has identified guided growths as a minimally invasive, safe and effective treatment options for CP patients with hip dysplasia. Most patients in the reported studies [32,34,47] were between 4 and 12 years of age and had a GMFCS of III–V; however, TMH-PF was performed in GMFCS I and II children by others [46]. It therefore remains controversial to apply guided growths procedures to ambulating patients, since their natural history of hip migration differs from GMFCS IV and V patients, and MP in GMFCS I patients commonly resolves spontaneously [52,53]. However, the progression of MP and late hip dislocation was reported in ambulating CP patients, with leg-length discrepancy, scoliosis, pelvic obliquity or deteriorating gait patterns being risk factors for poor outcome [54]. This can justify extended hip surveillance into adulthood and surgical intervention in selected cases [55]. Apart from GMFCS, Davids recommends an MP of 25 to 50% and an age between 4 and 10 years as indications for this guided growth procedure, and it was hypothesized that early surgical treatment is associated with greater potential for improvement of hip valgus; however, the likelihood of screw revision surgery due to the physis growing off the screw (whereby the screw no longer crosses the physis) is also increased [35]. A recent publication from the Cerebral Palsy Integrated Pathway Scotland (CPIPS) database concluded that the ‘point of no return’ for hip subluxation in this population was a MP > 46%, making spontaneous improvement unlikely [56]; however, others advocated a lower threshold for surgical intervention [12]. Furthermore, it was suggested that TMH-PF might be less effective in patients with an excessive (>50%) MP [45].

Implant choice varied considerably amongst all authors, ranging from 4.5 to 7.0 mm screw, both fully or partially threaded; however, it remains unclear if complications, including the screw backing out, are associated with the implant choice. Furthermore, it was recommended to pass two or three screw threads past the physis into the epiphysis [57], and Hsu et al. [48] have concluded that a centered screw position within the physis is associated with a reduced risk for physis growing off. The authors have suggested a centred screw position in young children, where early re-operation surgery i.e. due to growing off the physis is undesirable. In contrast, a more eccentrically placed screw near the medial physeal border is advised in older children, nearing skeletal maturity. Furthermore, the cox analysis revealed that an increased preoperative HSA was associated with higher rates of the screw growing off the physis. Hsieh et al. reported a combination of guided growth with simultaneous adductor tendon release [46].

The results of this meta-analysis are comparable to guided growth procedures performed for dysplasia of the hip (DDH) in smaller case series reported by other authors [33,49,58], where improvements of the femoral alignment and center edge angle (CEA) were reported after 2 years of treatment. Agus et al. [31] have trialed hip hemiepiphysiodesis procedures in DDH without the use of an implant; in order to avoid the need for screw revisions, the authors drilled the proximal medial physis in a small case series and found significantly improved physeal inclinations during follow-up. 

Our study has several limitations. Firstly, our meta-analysis is based on four small case series with high heterogeneity of the included patient characteristics (including age, GMFCS levels and length of follow-up). Guided growths of the proximal femur are, however, a relatively novel treatment, especially within the subgroup of cerebral palsy patients. Secondly, we noticed the poor quality of all studies included during the ROBIN-1 assessment. Thirdly, outcome measures and follow-up intervals were varied, and we pooled some outcomes to allow for further assessment during the meta-analysis, which can lead to an overestimation of the effect sizes reported. Hsieh et al. [46] included the outcomes of patients up to 12 years of age at the time of surgery and with a mean follow-up of 50 months (range 25–72 months); however, in the absence of long-term follow-up studies, it remains unclear how guided growths alter hip anatomy in the long term, including beyond skeletal maturity.

In conclusion, we have performed the first systematic review on the guided growth of the proximal physis in children with cerebral palsy. This novel and minimally invasive procedure has been shown to be safe and effective in the modulation of the proximal physis to correct coxa valga deformities, which can prevent progressive subluxation of the hip joint and may prevent the requirement for complex open-hip reconstruction surgery in this vulnerable cohort. Depending on the treatment duration and patient age, physis growing off the screw is a common complication, and patients and caregivers need to be counselled that screw revision is needed in about 50% of cases. Invasive pelvis reconstructions and femur osteotomies may be needed in only 5–21% of patients initially treated with guided growth, as reported in small cohort studies with short-term follow-ups, and long-term studies are needed to investigate appropriate indications and limitations of TMH-PF, including in ambulating patients or when combined with soft tissue releases [34,46].

## Figures and Tables

**Table 2:** Study characteristics and reported outcomes of CP patients undergoing TMH-PF surgery.

a. Study Characteristics.										
Reference	Study Design	Time Frame for Inclusion	Number of Hips in Number of Patients	Age at Surgery	GMFCS Level		Mean Follow-Up	Method of Fixation	Concomitant Soft Tissue Releases	Concomitant Botox Injections
[32]	Retrospective case series	January 2004–May 2012	13 hips in 9 patients	Mean 6.2 years (range 4.1–9.3 years)	IV V	6 patients 3 patients	45.6 months (range 24–96 months)	7.0 mm partially threaded Synthes screw	9/9 patients (common locations were psoas, adductor longus, gracilis and hamstrings)	0/9 patients
[34]	Retrospective case series	January 2007–December 2010	56 hips in 28 patients	Mean 7.5 years (range 4–11 years)	III IV V	7 patients 9 patients 12 patients	Not mentioned	4.5 mm partially threaded titanium Synthes screw	22/28 patients (bilateral distal hamstring lengthening	3/28 patients (medial hamstrings and adductors)
[46]	Retrospective case series	January 2012–December 2016	48 hips in 24 patients	Mean 8 years (range 5–12 years)	I II III IV V	3 patients 4 patients 7 patients 7 patients 3 patients	Mean 50 months (range 25–72 months)	6.0 mm fully threaded Acutrak, Acumed screw / 7.0 stainless steel, partially threaded, Synthes screw	24/48 hips 12/24 patients (adductor tenotomy)	0/24 patients
[47]	Retrospective case series	July 2012–September 2017	61 hips in 32 patients	Group 1– Median age 7 years (interquartile range 6.5–9.0) Group 2– Median age 7.5 years (interquartile range 6.0–9.0)	I II III IV V	4 patients 6 patients 10 patients 9 patients 3 patients		6.0 mm fully threaded Acutrak, Acumed screw / 7.0 stainless steel, partially threaded, Synthes screw	Not described	Not described
**b. Reported Outcomes.**										
**Reference**			**Preoperative Radiographic Measurements**	**Radiographic Measurements at 3 Months**	**Radiographic Measurements at 6 Months**	**Radiographic Measurements at 1 Year**	**Radiographic Measurements at 2 Years**	**Radiographic Measurements at Final Follow-Up**	**Number of Hips Growing Off Screws**	**Revision Surgery**
[32]								Final follow-up at 5.8 years		
		MP	52.2% (range 36–83%)	45.8% (*p* = 0.012)		40.3% (*p* = 0.016 *)	37.1% (*p* = 0.021 *)		13/13 hips (100%)	
		HSA		173.3°		166.4° (*p* < 0.001 *)	162.7° (*p* = 0.15 *)	157.2°		
[34]								Final follow-up at 5 years		
		MP	33.5% (±11.29%)		29.23% (*p* < 0.001)	25.96% (*p* < 0.001 †)		23.16% (*p* < 0.001†)	9/56 hips 6/28 patients	6 screw revisions 3 subsequent VDROs
		NSA	156° (±10°)		150° (*p* < 0.001)	146° (*p* < 0.001 †)		142° (*p* < 0.001 †)		
		AI	23° (±6°)		20° (*p* < 0.001)	18° (*p* < 0.001 †)		17° (*p* < 0.001 †)		
[46]								Final follow-up at a mean of 50 months		
		HSA	163° (±6°)					150° (*p* < 0.001 †)		
		HEL	10° (±4°)					25° (*p* < 0.001 †)		
		AI	22° (±6°)					19° (*p* < 0.001 †)		
		MP	39% (±10%)					29% (*p* < 0.001 †)		
[47]		HSA	Group 1–163.6° Group 2–161.8°				Group 1–149.7° (*p* < 0.001 †) Group 2–153.1° (*p* < 0.001 †)		Group I– 16/37 hips Group 2– 4/24 hips	
		MP	Group 1–28.7% Group 2–29.0%				Group 1–23.8% (*p* < 0.001 †) Group 2–27.5% (*p* < 0.265 †)			
		AI	Group 1–21.0° Group 2–21.2°				Group 1–19.4° (*p* < 0.001 †) Group 2–19.8° (*p* < 0.010 †)			
		FAVA	Group 1–32.0° Group 2–31.2°				Group 1–24.3° (*p* < 0.001 †) Group 2–24.9° (*p* < 0.001 †)			

HEL–Hilgenreiner’s epiphyseal angle; * *p*-value calculated by comparison to previous radiographic measurement; † *p*-value calculated by comparison to preoperative radiographic measurement.
